# The Impact of Curcumin, Resveratrol, and Cinnamon on Modulating Oxidative Stress and Antioxidant Activity in Type 2 Diabetes: Moving beyond an Anti-Hyperglycaemic Evaluation

**DOI:** 10.3390/antiox13050510

**Published:** 2024-04-24

**Authors:** Michalina Banaszak, Ilona Górna, Dagmara Woźniak, Juliusz Przysławski, Sławomira Drzymała-Czyż

**Affiliations:** 1 Department of Bromatology, Poznan University of Medical Sciences, Rokietnicka 3, 60-806 Poznan, Poland; dagmara.wozniak@student.ump.edu.pl (D.W.); jotespe@ump.edu.pl (J.P.); drzymala@ump.edu.pl (S.D.-C.); 2 Doctoral School, Poznan University of Medical Sciences, Bukowska 70, 60-812 Poznan, Poland

**Keywords:** antioxidants, dietary supplements, phytochemicals, total antioxidant capacity, interleukin 6, tumour necrosis factor α

## Abstract

Research indicates that up to half of the population resorts to dietary supplements for managing diseases such as type 2 diabetes rather than changing their nutritional habits. These supplements not only aim to have an anti-hyperglycaemic effect but also seek to reduce oxidative stress to prevent diabetes complications. This systematic literature systematic review aims to evaluate the efficacy of curcumin, resveratrol, and cinnamon in modulating oxidative stress and antioxidant activity in individuals with type 2 diabetes. Data were collected from PubMed, Web of Sciences, and Scopus databases regarding the impact of curcumin, resveratrol, and cinnamon on total antioxidant capacity (TAC), malondialdehyde (MDA), tumour necrosis factor α (TNF-α), interleukin 6 (IL-6), and high-sensitivity C-reactive protein (hs-CRP) levels for this review. Effect sizes for each study were calculated using Cohen’s or Hedges’s d coefficient. Parameters of oxidative stress and inflammatory status, such as TAC, MDA, TNF-α, IL-6, and hs-CRP, improved following phytochemicals. Additionally, curcumin, resveratrol, and cinnamon exhibited regulatory effects on carbohydrate metabolism by reducing glucose, insulin, and glycated haemoglobin concentrations and lipid metabolism by lowering total cholesterol (TC), low-density lipoprotein (LDL), and triglycerides (TG) and increasing high-density lipoprotein (HDL). Incorporating curcumin, resveratrol, and cinnamon into diets may be beneficial for maintaining organism homeostasis and improving metabolic control in individuals with type 2 diabetes. However, the conflicting results reported in the literature highlight the need for further detailed investigations into the effectiveness of phytochemical use for type 2 diabetes.

## 1. Introduction

Dietary supplements are used by billions of people daily, with an estimated 22% to 53% of the population taking dietary supplements, particularly multivitamin preparations [1]. A dietary supplement is a food product designed to supplement nutritional deficiencies, but the definition may vary depending on the country. These products can come in tablets, capsules, coated tablets, gummies, water-soluble powders, or drops. Furthermore, their content has no restrictions, ranging from vitamins and minerals to herbs, but they cannot have medicinal properties [1,2]. In the European Union, dietary supplements are approved by institutions responsible for introducing new food products to the market. They do not require prior clinical trials, making the composition and potential complications from using a specific supplement unclear. The US market treats dietary supplements even more liberally; they do not need to be reported anywhere and only adverse side effects can lead to the withdrawal of a product from the market [3]. A proper balanced diet should provide all the essential macro- and micronutrients needed for the body’s functioning. Supplementation should ideally be conducted under the supervision of a doctor in specific cases (Table 1).

Non-communicable diseases (NCDs) represent a significant global challenge, with World Health Organization data (WHO, 2019) indicating that they are responsible for 74% of deaths worldwide, translating into 41 million deaths annually. The highest number of deaths is attributed to cardiovascular diseases (CVDs) (17.9 million annually), followed by cancers (9.3 million annually), chronic respiratory diseases (4.1 million annually), and diabetes (2.0 million annually, including diabetic chronic kidney disease). Consequently, searching for new methods of preventing and treating NCDs should be an essential element of global health strategies aimed at improving people’s quality of life and reducing the burden on healthcare systems [6].

This literature review focuses on type 2 diabetes (T2DM), a metabolic disorder characterised by improper insulin functioning resulting in elevated blood glucose levels (hyperglycaemia). T2DM is often accompanied by other disorders, including insulin resistance and hyperlipidaemia. Prolonged hyperglycaemia leads to serious complications, including nephropathy, neuropathy, and retinopathy, as well as micro- and macroangiopathy. Individuals with T2DM have a higher risk of cardiovascular and cancer complications, as well as the development of chronic inflammatory conditions. Diabetic complications are the leading cause of death in individuals with diabetes, making prevention crucial. T2DM should be treated pharmacologically and through diet and lifestyle changes. The primary goal of therapy is to maintain optimal metabolic control and reduce excess body weight to maintain a desirable body mass [7,8,9,10]. People with T2DM often struggle to change dietary habits, so they readily turn to dietary supplements to manage the disease [11,12].

There is increasing evidence that active substances derived from plants (phytochemicals), such as carotenoids, polyphenols, isoprenoids, phytosterols, and some polysaccharides, are crucial for health. Products containing phytochemicals are referred to as functional foods and their consumption may lower the risk of developing cardiovascular diseases, neurodegenerative diseases, type 2 diabetes, etc. [13]. They possess antioxidant, anti-inflammatory, antimicrobial, anti-cancer, anti-rheumatic, immunomodulatory, and anti-hyperglycaemic properties [14,15]. Chronic low-grade inflammation and accompanying metabolic disorders play a crucial role in T2DM and associated complications. Studies show that individuals with T2DM experience increased oxidative stress due to elevated reactive oxygen species activity and/or decreased antioxidant defence mechanisms. Chronic high blood glucose levels induce oxidative stress, leading to impaired pancreatic β cell functioning, exacerbating insulin resistance, and promoting vascular complications [16].

This systematic literature review evaluates the effectiveness of curcumin, resveratrol, and cinnamon in modulating oxidative stress and antioxidant activity when used as single-component dietary supplements. Summarising the current knowledge about the impact of selected phytochemicals on the health of individuals with T2DM can contribute to expanding existing therapeutic possibilities.

## 2. Materials and Methods

**Figure 1:**
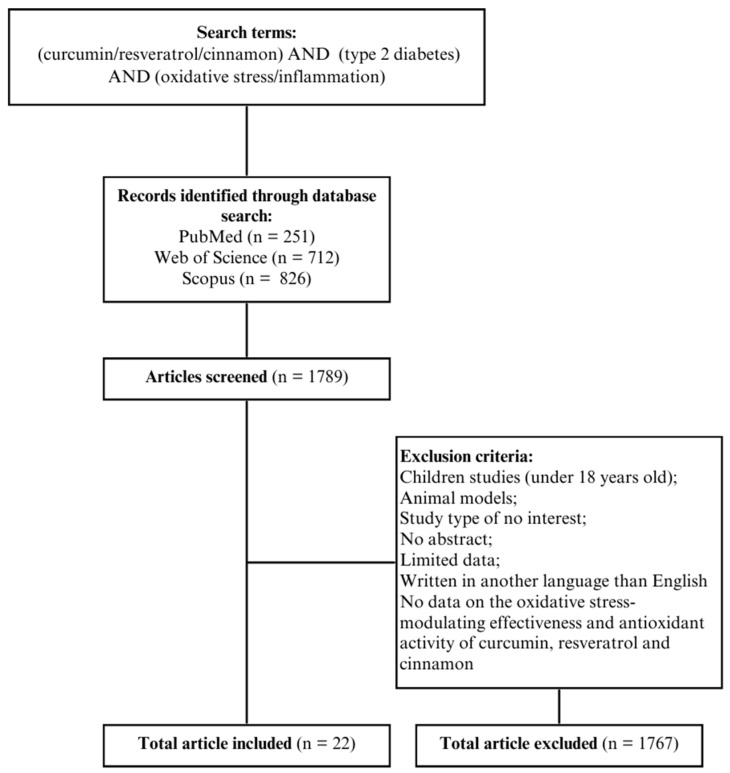
Search strategy.

The review included randomised controlled trials and descriptive studies from the last 10 years available in the PubMed, Web of Sciences, and Scopus databases. The included studies were conducted on adults (>18 years old), written in English, and focused on the impact of curcumin, resveratrol, and cinnamon on total antioxidant capacity (TAC), malondialdehyde (MDA), tumour necrosis factor α (TNF-α), interleukin 6 (IL-6), and high-sensitivity C-reactive protein (hs-CRP). Animal studies, research involving children and adolescents, systematic reviews, and meta-analyses were excluded from the review. Effect sizes for each study (where necessary data were available) were calculated using Cohen’s or Hedges’s d coefficient. The search strategy is presented in Figure 1.

## 3. The Assessment of Preparations Containing Curcumin

Curcumin is an active chemical compound present in the rhizome of the plant Curcuma longa, also known as turmeric. It exhibits antioxidant, anti-inflammatory, antimicrobial, anticancer, anti-rheumatic, immunomodulatory, anti-hyperglycaemic, and cardio-renal-hepato-protective properties. In one animal study, curcumin and its analogues were shown to have a similar mechanism of action to thiazolidinedione, an antidiabetic drug, through the activation of the peroxisome proliferator-activated receptor gamma (PPAR-γ), suggesting that curcumin may be effective in regulating glycaemia and lipid levels [15]. Curcumin appears to have beneficial effects in reducing fasting glucose, glycated haemoglobin (HbA_1c_), homeostatic model assessment of insulin resistance (HOMA-IR), and TNF-α. It also positively influences lipid metabolism by lowering low-density lipoprotein (LDL) cholesterol and triglyceride levels while increasing high-density lipoprotein (HDL) cholesterol. Additionally, most studies have tested various curcumin formulations, especially in combination with other bioactive substances (e.g., piperine), and studies evaluating pure curcumin are limited [17] (Table 2).

Curcumin demonstrates beneficial effects not only on the glycaemic control but also on anthropometric parameters. Hodaei et al. [18] recruited 53 individuals with excess body weight and T2DM. Participants were divided into a study group receiving 1500 mg/day of curcumin and a control group receiving a placebo three times a day. After 10 weeks of supplementation, the study group showed a significant decrease in body weight, body mass index (BMI), waist circumference, and blood glucose levels (*p* < 0.05) compared to the control group. However, there were no changes in HbA_1c_, insulin, MDA, TAC, HOMA-IR, and pancreatic beta-cell function (HOMA-B). These studies do not confirm the beneficial impact of curcumin on oxidative stress.

Chronic kidney disease (CKD) is a serious complication of T2DM. Oxidative stress accompanying proteinuria in kidney disease without diabetes or in diabetic patients may accelerate CKD progression. Jiménez-Osorio et al. [19] investigated the impact of curcumin supplementation on markers of oxidative stress, antioxidant enzyme activity, and nuclear factor erythroid 2-related factor 2 (Nrf2) activation. Depending on the cause of CKD, patients were divided into four groups: CKD with non-diabetic proteinuria receiving a placebo (*n* = 26) or curcumin at a dose of 320 mg/day (*n* = 24), and CKD with diabetic proteinuria receiving a placebo (*n* = 23) or curcumin at a dose of 320 mg/day (*n* = 28). The intervention lasted 8 weeks, but curcumin supplementation did not improve proteinuria, estimated glomerular filtration rate, or lipid profile. However, in individuals with non-diabetic proteinuria and CKD, curcumin attenuated lipid peroxidation (*p* < 0.05), whereas curcumin increased antioxidant capacity in individuals with T2DM and CKD with proteinuria (*p* < 0.05). No impact of curcumin supplementation on antioxidant enzyme activity or Nrf2 activation was observed. These results suggest a potential reduction in oxidative stress in patients with diabetes and CKD.

Yang et al. [20] obtained results different from those mentioned above. Patients with T2DM and CKD (*n* = 14) received 500 mg/day of curcumin for 15 days (30 days in patients with overt proteinuria). Curcumin supplementation significantly reduced urinary microalbumin excretion and lowered serum MDA levels by enhancing the specifically Nrf2-regulated protein, NAD(P)H quinone oxidoreductase 1 (NQO-1), along with other antioxidant enzymes in the lymphocytes. Additionally, patients showed reduced serum lipopolysaccharide (LPS) content and increased inhibitor of nuclear factor kappa-B kinase (IκB kinase) proteins inhibiting inflammatory signalling in lymphocytes. Interestingly, curcumin stimulated the activity of several gut bacteria crucial for maintaining the integrity and functioning of the gut barrier. In summary, short-term curcumin intervention inhibits the progression of DKD by activating the Nrf2 antioxidant system and exerting anti-inflammatory effects in patients with T2DM. However, the study has some limitations, such as a small number of participants, lack of a control group, and short supplementation duration. The obtained results are optimistic but need confirmation in a larger patient population.

The impact of physical exercise, as a component of a healthy lifestyle, on the metabolic state and oxidative stress parameters was compared with curcumin supplementation in individuals with hyperlipidaemia and T2DM. Forty-two individuals were randomly assigned to four groups: aerobic training + curcumin (*n* = 11), aerobic training + placebo (*n* = 10), curcumin supplementation (*n* = 11), and control + placebo (*n* = 10). The training involved 20–40 min of exercise daily, three days a week for eight weeks (60–75% of maximum heart rate (HRmax)). Patients assigned to the curcumin supplementation groups received 700 mg/day for 8 weeks. In all three intervention groups, hs-CRP levels decreased compared to in the control group (*p* < 0.05). The aerobic training + curcumin supplementation group showed the best results with significantly reduced MDA (*p*  =  0.001; *p*  =  0.001) and hs-CRP (*p*  =  0.028; *p*  =  0.041) levels and increased glutathione (GSH) levels (*p*  =  0.003; *p*  =  0.001) and TAC (*p*  =  0.001; *p*  =  0.001) compared to the aerobic-training and curcumin-supplementation-alone ones, respectively. These results demonstrate the positive effects of curcumin supplementation and physical activity on the metabolic state, oxidative stress markers, and hs-CRP [21].

Curcumin supplementation appears to have beneficial effects in individuals with T2DM and coronary heart disease (CHD). Shafabakhsh et al. [22] recruited 60 patients with T2DM and CHD aged 45–85 and were randomly assigned to two groups—the experimental group receiving 1000 mg/day of curcumin and the control group receiving a placebo for 12 weeks. After the intervention, the experimental group showed a significant reduction in MDA levels (*p* = 0.01) and a significant increase in TAC (*p* = 0.04) and GSH levels (*p* = 0.001) compared to those of the placebo group. Additionally, curcumin consumption increased the level of peroxisome proliferator-activated receptor gamma (PPAR-γ) (*p* = 0.01). Interestingly, curcumin supplementation significantly decreased the Pittsburgh Sleep Quality Index (PSQI) (*p* = 0.01) compared to that of the placebo group, indicating the pleiotropic effects of curcumin in humans.

Research suggests that reducing adipocyte fatty acid-binding protein (A-FABP) levels in serum may be associated with improved metabolic parameters in individuals with T2DM. Na et al. [23] investigated whether curcumin supplementation reduced A-FABP levels and verified the impact of curcumin on oxidative stress and inflammatory biomarkers. One hundred individuals with T2DM were randomly assigned to an experimental group (*n* = 50) receiving curcumin (300 mg/day) and a control group (*n* = 50) receiving a placebo. The intervention lasted for 3 months. The curcumin-receiving group had significantly reduced serum A-FABP levels (*p* < 0.001), CRP (*p* < 0.001), TNF-α (*p* = 0.047), and IL-6 (*p* < 0.001) compared to the placebo one. Additionally, curcumin supplementation also significantly increased the activity of superoxide dismutase (SOD) in serum (*p* < 0.005) but did not affect GSH activity and MDA concentration. The researchers concluded that curcumin may have antidiabetic effects by reducing A-FABP levels in serum.

Curcumin also exhibits lipid-metabolism-lowering effects. Forty-four individuals with T2DM were randomly assigned to a group supplementing with curcumin at a dose of 1500 mg/day or to a control group receiving a placebo for 10 weeks. The average serum triglyceride (TG) level decreased in the experimental group compared to the baseline (*p* < 0.05). In the curcumin-supplementing group, the average hs-CRP concentration significantly decreased (*p* < 0.05), and the average adiponectin concentration significantly increased (*p* < 0.05) compared to in the placebo group. These results suggest that curcumin intake may reduce diabetic complications by decreasing TG levels and inflammatory markers [24].

Similar results were obtained by Shafabaksh et al. [25]. However, they administered nanocurcumin at a dose of 80 mg/day (experimental group, *n* = 30) for 12 weeks to individuals with T2DM undergoing haemodialysis. The remaining participants were assigned to the control group receiving a placebo (*n* = 30). Supplementation significantly reduced the fasting blood glucose level (*p* < 0.05), serum insulin (*p* < 0.05), TG (*p* < 0.05), very-low-density lipoprotein (VLDL) (*p* < 0.05), total cholesterol (TC) (*p* < 0.05), LDL (*p* < 0.05), and the ratio of TC to HDL (*p* < 0.05) compared to the placebo. Nanocurcumin also significantly reduced hs-CRP (*p* < 0.05) and MDA (*p* < 0.05) but significantly increased TAC (*p* < 0.05) and the total nitrate level (*p* < 0.001) in the experimental group compared to in the placebo group.

Similar methods were applied in the study by Dastani et al. [26] of patients with T2DM and mild-to-moderate coronary artery disease (CAD) (<70% stenosis in angiography) (*n* = 64). The participants were randomly assigned to receive nanocurcumin (80 mg/day), a placebo, and optimal medications for 90 days. Nanocurcumin supplementation significantly decreased hs-CRP and lipoprotein A (*p* < 0.001 and *p* = 0.043, respectively), as well as the mean per cent change (%Δ) in levels of hs-CRP and lipoprotein A (*p* < 0.001 and *p* = 0.007, respectively) compared to those of the control group. The researchers concluded that nanocurcumin at a dose of 80 mg/day might prevent atherosclerosis progression and further cardiovascular events in patients with T2DM and CAD by reducing the inflammatory marker hs-CRP.

These analyses raise concerns regarding the doses of supplements used. According to the European Food Safety Authority (EFSA) recommendations, the daily dose of curcumin should not exceed 3 mg/kg body weight/day (on average, about 180–240 mg/day). Some of the publications included in this review were not conducted in Europe, but it seems that using doses in the 1–2 g/day range may be unsafe for health [27].

## 4. The Assessment of Preparations Containing Resveratrol

Resveratrol (3,4′,5-trihydroxy stilbene) is a natural polyphenol in red wine, grapes (especially in the skins of red grapes), berries, peanuts, and chocolate [28]. It has a positive impact on the oxidative–antioxidative balance by increasing the expression of antioxidant enzymes, including glutathione peroxidase, SOD, catalase, and heme oxygenase-1 (HO-1). It also regulates various signalling pathways, including sirtuin 1 (SIRT1), Nrf2, and the nuclear factor kappa-light-chain-enhancer of activated B cells (NF-κB) to enhance GSH levels and maintain cellular redox balance [29,30]. Evidence collected in meta-analyses confirming the anti-hyperglycaemic effects of resveratrol in the treatment of T2DM is contradictory [31,32]. More research is needed to fully assess its usefulness in supporting T2DM treatment, especially in regulating oxidative stress (Table 3).

Mahjabeen et al. [33] investigated the impact of resveratrol supplementation on carbohydrate metabolism parameters, oxidative stress, inflammation, and miRNA expression in individuals with T2DM. One hundred and ten participants were randomly assigned to the resveratrol group (*n* = 55; 200 mg/day) and placebo group (*n* = 55) for 24 weeks. After the intervention, the resveratrol group showed a significant reduction in glucose (*p* = 0.016), insulin (*p* = 0.001), HOMA-IR (*p* = 0.001), MDA (*p* = 0.001), hs-CRP (*p* = 0.046), TNF-α (*p* = 0.001), and IL-6 (*p* = 0.001) compared to the control group. Patients receiving resveratrol exhibited a twofold decrease in the expression of miRNA-34a, miRNA-375, miRNA-21, miRNA-192 and an increase in the expression of miRNA-126 and miRNA-132 compared to those receiving the placebo. These results confirm the beneficial effects of resveratrol on various markers, and specific miRNA fragments may serve as new biochemical markers in diagnosing and treating T2DM.

The antioxidant effect of resveratrol was also verified by Seyyedebrahimi et al. [34] in a study of 46 individuals with T2DM randomly assigned to the experimental group (800 mg/day of resveratrol) or the control group (placebo). Participants took capsules for 2 months. Resveratrol supplementation reduced the serum protein carbonyl content (*p* = 0.007), superoxide anion (O_2_^•−^) levels in peripheral blood mononuclear cells (PBMCs), and significantly increased TAC (*p* = 0.002) and the total thiol content in the serum (*p* = 0.01) compared to the placebo. The expression of Nrf2 (*p* = 0.047) and SOD (*p* = 0.005) also increased in the group supplemented with the phytochemicals. Resveratrol had no significant impact on the metabolic and anthropometric parameters, except for a significant reduction in body weight, BMI (*p* = 0.006), and blood pressure levels (systolic *p* = 0.002 and diastolic *p* = 0.000).

García-Martínez et al. [35] evaluated the antioxidant effectiveness of resveratrol based on dosage. Ninety-seven individuals with T2DM were assigned to three groups: 1000 mg/day of resveratrol (*n* = 37), 500 mg/day of resveratrol (*n* = 32), and placebo (*n* = 28). Biochemical parameters, oxidative stress, and SIRT1 levels were measured at the beginning of the study and after 6 months. There was a significant increase in TAC, the antioxidant gap, the percentage of individuals without oxidative stress, and SIRT1 (*p* < 0.05) in the group taking 1000 mg/day of resveratrol. Meanwhile, lipid peroxides, isoprostanes, and CRP levels significantly increased (*p* < 0.05) in the control group. These results suggest that consuming 1000 mg/day of resveratrol has a more effective antioxidant effect than 500 mg/day, coinciding with a statistically significant increase in SIRT1 levels in adults with T2D.

T2DM frequently affects older individuals, and as such, some studies are based on this population. In the study by Ma et al. [36], 472 elderly individuals with T2DM were randomly assigned to the experimental group (resveratrol at a dose of 500 mg/day; *n* = 242) and the control group (placebo; *n* = 230). After 6 months of supplementation, there was a significant improvement in glucose metabolism (*p* < 0.05), insulin tolerance (*p* = 0.005), and insulin metabolism (*p* < 0.05) in individuals taking resveratrol compared to those taking the placebo. The experimental group also showed a significant reduction in the production and activity of glucose-6-phosphatase (G6Pase), hs-CRP (*p* = 0.038), HbA_1c_ (*p* = 0.034), IL-6 (*p* = 0.043), TNF-α (*p* = 0.003), and IL-1β (*p* = 0.002) compared to the placebo group. Resveratrol supplementation lowered blood glucose parameters (*p* < 0.05), positively influenced lipid profile parameters (TC, LDL, HDL, TG) (*p* < 0.05), and affected kidney function compared to the placebo, suggesting that resveratrol may be a potential therapeutic agent in treating elderly individuals with T2DM.

Bo et al. [37] examined the dose-dependent effects of resveratrol on CRP and the improved metabolic parameters in patients with T2DM. Participants were randomly assigned to receive 500 mg/day (*n* = 65) or 40 mg/day (*n* = 65) resveratrol or placebo (*n* = 62) for 6 months. A dose-dependent, albeit non-significant, decrease in CRP (5.6% in the 40 mg/day group; *p* = 0.78 and 15.9% in the 500 mg/day group; *p* = 0.15) compared to the placebo was demonstrated. No significant differences were observed in body weight, BMI, waist circumference, blood pressure values, fasting glucose, HbA_1c_, insulin, C-peptide, free fatty acids (FFAs), liver aminotransferases, uric acid, adiponectin, and IL-6, but TC (*p* = 0.01) and TG (*p* = 0.05) slightly increased in individuals receiving 500 mg/day of resveratrol.

Similar results were obtained by Khodabandehloo et al. [38], who demonstrated that resveratrol supplementation does not improve inflammatory markers in individuals with T2DM. Twenty-five patients with T2D received resveratrol supplementation at 800 mg/day, while twenty individuals received a placebo for 8 weeks. The study did not show any significant differences in the percentage of CD14+ and CD16+ monocytes, LPS-induced cytokine secretion, serum levels of inflammatory cytokines, or the expression of inflammatory genes in the resveratrol group compared to in the placebo one. Additionally, no significant changes were observed in metabolic and anthropometric parameters in the study group, except for a significant reduction in fasting blood glucose levels (*p* = 0.048) and blood pressure (systolic *p* = 0.002 and diastolic *p* = 0.006).

Bashmakov et al. [39] also observed no changes in CRP values in their study. Twenty-four patients with T2DM and newly diagnosed diabetic foot ulcers were divided into two groups: an experimental group receiving 100 mg/day of trans-resveratrol (*n* = 14) and a control group receiving a placebo (*n* = 10) for 60 days. Patients taking resveratrol showed a reduction in parameters reflecting the depth of diabetic ulcer compared to those taking the placebo. Additionally, they also exhibited slightly better results in the foot pressure test. The experimental group showed a significant decrease in serum fibrinogen levels but not in CRP. Supplementation with trans-resveratrol contributes to reducing the size of foot ulcers and may be used as a supportive measure in the treatment of diabetic foot.

Periodontal diseases are chronic conditions associated with T2DM. Interestingly, resveratrol has antimicrobial effects against periodontal pathogens like *A. actinomycetemcomitans* and *P. gingivalis*. The body’s response to these microorganisms can increase the production of inflammatory markers, including IL-6, TNF-α, and IL-1β [41]. Therefore, Javid et al. [40] determined the impact of resveratrol on inflammatory markers in patients with T2DM and periodontal diseases. Fifty individuals with T2DM and chronic periodontitis were randomly assigned to either the intervention group (resveratrol 480 mg/day; *n* = 25) or the control group (placebo—starch; *n* = 25) and received resveratrol supplements or a placebo for 4 weeks. Resveratrol supplementation significantly reduced the mean serum IL-6 concentration (*p* = 0.039) compared to the baseline, with no significant differences between the two groups in the average levels of IL6, TNF-α, TAC, and a key periodontal marker—clinical attachment loss (CAL)—after the intervention.

## 5. The Assessment of Preparations Containing Cinnamon

Cinnamon extracts contain polyphenols such as catechin, procyanidin, cinnamic acid, and flavones (cinnamaldehyde and trans-cinnamaldehyde). Cinnamon interacts with hepatic glucose homeostasis by suppressing phosphoenolpyruvate carboxykinase (PEPCK) and G6Pase, which control gluconeogenesis [42]. Available studies show that cinnamon supplementation in T2DM reduces fasting glucose levels, HOMA-IR, TC, LDL, and TG with no significant impact on HbA_1c_ [43,44]. Few studies have assessed the influence of cinnamon extract supplementation on oxidative stress and inflammatory parameters, which could potentially reduce the risk of T2DM complications [45] (Table 4).

Sahib [46] assessed the impact of cinnamon supplementation on carbohydrate metabolism and oxidative stress parameters in poorly controlled T2DM. Twenty-five individuals with T2DM, treated exclusively with a sulfonylurea derivative (glibenclamide), were randomly assigned to either the experimental group receiving 1g/day of cinnamon or the control group receiving a placebo for 12 weeks. The cinnamon supplementation group showed a significant reduction in fasting blood glucose compared to the baseline and the placebo group (*p* ≤ 0.001). Despite a decrease in HbA_1c_, the result was not statistically significant. In the experimental group, serum GSH levels significantly increased, while serum MDA levels significantly decreased compared to the baseline and in the placebo group (*p* ≤ 0.001). Additionally, cinnamon supplementation led to a significant increase in SOD levels (*p* ≤ 0.05). In summary, taking cinnamon as an adjunct to antidiabetic medications may be beneficial in the treatment of poorly controlled type 2 diabetes.

Azimi et al. [47] investigated the impact of four herbs as complementary treatments in T2DM. In this clinical study, 204 individuals with T2DM were randomly assigned to four intervention groups receiving three cups of black tea and 3 g/day of cardamom, cinnamon, or ginger, or 1 g/day of saffron, and one control group that consumed only three cups of tea without any herbal medications for 8 weeks. Supplementation with all four herbs significantly affected total cholesterol, LDL, and HDL levels (*p* < 0.05) compared to those of the control group. There was no significant impact on glycaemic control measures, anthropometry, inflammation, and oxidative stress. Cinnamon intake significantly reduced fasting blood sugar levels (*p* = 0.003) and hs-CRP (*p* = 0.04) in intra-group comparisons, while ginger intake significantly decreased F2-isoprostane levels (*p* = 0.01) and hs-CRP (*p* = 0.04).

Davari et al. [48] assessed the impact of cinnamon supplementation on serum levels of NF-kB, SIRT1, hs-CRP, IL-6, and TNF-α in individuals with T2DM. Forty-four adults with T2DM were randomly assigned to the intervention group (3g/day of cinnamon; *n* = 22) and the control group (placebo; *n* = 22) for 8 weeks. No significant differences were observed in hs-CRP levels (*p* = 0.29), TNF-α (*p* = 0.27), IL-6 (*p* = 0.52), and SIRT1 (*p* = 0.51) between the groups. Interestingly, the average level of hs-CRP significantly decreased in the placebo group (*p* = 0.008). The results suggest that cinnamon supplementation in T2DM does not benefit reducing the levels of parameters that play a significant role in atherogenesis.

Mirmiran et al. [49] evaluated cinnamon supplementation in the context of preventing atherosclerosis. Individuals with T2DM were randomly assigned to the intervention group (3g/day of cinnamon; *n* = 22) and the control group (placebo; *n* = 22) for 8 weeks. There was a significant reduction in the mean levels of ICAM-1 and VCAM-1 in both the cinnamon supplementation and the placebo groups compared to the baseline (*p* < 0.001). Additionally, no significant differences were observed between the groups regarding fasting glucose, fasting insulin, HbA_1c_, HOMA-IR, TAC, MDA, and carboxymethyl lysine (*p* > 0.05) after 8 weeks of intervention. Similar conclusions were reached by Talaei et al. [50] (presumably the same study population, same methods, and same results), indicating that cinnamon supplementation does not significantly impact oxidative stress parameters in individuals with T2DM.

## 6. Discussion

This review aimed to assess the impact of curcumin, resveratrol, and cinnamon on inflammatory and oxidative stress parameters in T2DM. Most studies reported the effects of TAC, MDA, TNF-α, IL-6, and hs-CRP. TAC is an oxidative stress parameter that assesses the number of free radicals captured by the test solution to evaluate the antioxidant capacity of biological samples [51]. MDA is also an important marker of oxidative stress, a three-carbon small-molecule aldehyde formed during the breakdown of peroxides resulting from the action of free radicals on polyunsaturated fatty acids. The degree of lipid peroxidation can be assessed based on the amount of MDA in tissues [52,53]. TNF-α is a major regulator of inflammatory responses and is involved in the development of pathological complications. It is mainly produced by activated macrophages, T lymphocytes, and NK cells, and to a much lesser extent by other tissues. TNF-α participates in the pathogenesis of some inflammatory and autoimmune diseases, triggering various inflammatory molecules, including other cytokines and chemokines [54] such as IL-6, a soluble mediator with pleiotropic effects on inflammation, immune response, and haematopoiesis. Its synthesis occurs in the local pathological changes in the initial phase of inflammation. Dysregulated continuous IL-6 synthesis has a pathological impact on chronic inflammation associated with NCDs [55]. Hs-CRP is a circulating acute-phase protein used as a marker of inflammation and participates in the immune response. Elevated CRP levels occur in infections, inflammatory conditions, autoimmune and inflammatory diseases, injuries, or cancers. Additionally, it is increasingly used to assess CVD risk [56,57]. At this stage, it is essential to emphasise that a significant limitation of this review is the lack of consistency in the analysed parameters (hence the impossibility of conducting a meta-analysis). The multitude of assays performed in individual manuscripts allows for a thorough investigation of the anti-inflammatory activity of the analysed phytochemicals. Still, the lack of a typical single marker complicates their interpretation.

Oxidative stress plays a crucial role in T2DM development. Hyperglycaemia induces oxidative stress, and oxidative stress, in turn, leads to an increase in blood glucose levels. Inflammatory cytokines play a significant role in the pathophysiology of diabetes complications. They are typically evaluated during fasting but also postprandially. Specifically, research indicates that consuming a high-fat meal increases TNF-α levels, contributing to endothelial dysfunction and oxidative stress. This vicious cycle can contribute to impaired pancreatic β-cell function, exacerbate insulin resistance, and intensify the development of diabetic complications [58,59,60].

**Figure 2:**
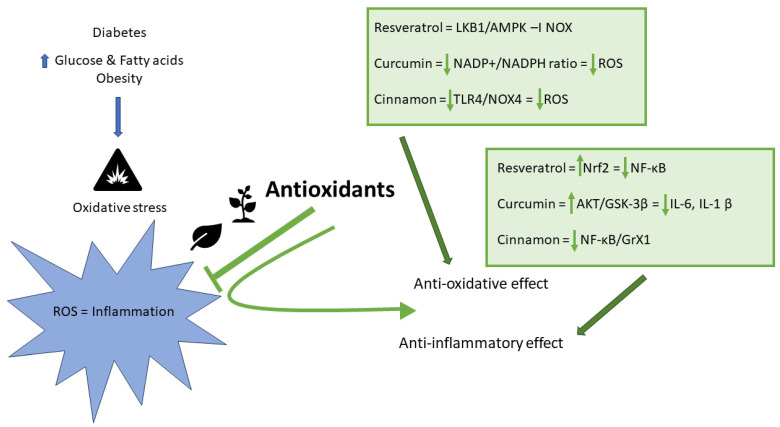
Selected physiological mechanisms of action of antioxidants in T2DM. ROS—reactive oxygen species; LKB1—Liver Kinase B1; AMPK—AMP–Activated Protein Kinase; AMP—Adenosine Monophosphate; NOX—Nicotinamide Adenine Dinucleotide Phosphate Oxidase; NADPH—Nicotinamide Adenine Dinucleotide Phosphate; TLR4/NOX4—Toll-like Receptor 4-NADPH Oxidase 4; Nrf2—nuclear factor erythroid 2-related factor 2; NF-κB—nuclear factor-κB; AKT—Protein Kinase B; GSK-3β—Glycogen Synthase Kinase 3β; IL-6—interleukin-6; IL-1β—interleukin-1β; GrX-1—Glutaredoxin-1.

The phytochemicals discussed in this review are antioxidants, substances that significantly delay or prevent the oxidation of substrates, and the primary function is to provide protectection from the adverse effects of free radicals (Figure 2) [51].

The described phytochemicals are of natural origin, meaning they are easily accessible from vegetables and fruits, so including them in diets can positively impact the metabolic control of diabetes and associated diseases. Furthermore, these active substances also have anti-hyperglycaemic effects, influencing lipid metabolism by reducing total cholesterol, LDL, and triglyceride concentrations and increasing HDL cholesterol levels. The main dietary supplement used by individuals with T2DM is white mulberry, a phytochemical with anti-hyperglycaemic properties. Due to numerous well-documented studies (23 meta-analyses and systematic reviews available in the Pub-Med database) demonstrating the anti-hyperglycaemic effectiveness of this phytochemical, it was excluded from this review as the intended focus was on demonstrating the effectiveness of selected phytochemicals in reducing oxidative stress parameters.

## 7. Conclusions

This literature review demonstrated that curcumin, resveratrol, and cinnamon exhibit anti-inflammatory and antioxidant effects, as evidenced by improving oxidative stress and inflammatory parameters such as TAC, MDA, TNF-α, IL-6, and hs-CRP. Considering the effect size calculated based on Cohen’s and Hedges’s tests, curcumin and resveratrol were the most effective. Given the conflicting results reported in the literature, the impact of phytochemicals on individuals with diabetes should undergo further detailed investigations. Further research could identify new therapeutic targets to alleviate inflammation, reducing the risk of cardiovascular diseases, nephropathy, neuropathy, and other complications often associated with T2DM. Although antioxidant supplementation, complemented by a balanced diet, may help maintain physiological homeostasis, it is critical to address persistent concerns regarding dietary supplements’ clinical effectiveness and safety. Despite their widespread use, a lack of scientific consensus exists regarding their therapeutic effectiveness. The phytochemicals we discuss are compounds of plant origin; they do not pose a threat to health or life. However, it has not been detected whether the long-term use of them in a synthetic form and at high doses potentially harms human health and life. It is recommended to exercise caution and seek a specialist’s opinion before taking supplementation.

## Figures and Tables

**Table 1:** Indications for the use of dietary supplements (own elaboration based on [2,4,5]).

Population	Nutrients
People following restrictive diets (<1600 kcal)	Vitamins and minerals, protein, omega-3 acids
Elderly	Vitamin B_12_, protein
Pregnant and lactating women	Folic acid, iodine, vitamin D_3_, iron, magnesium in indicated cases
People following exclusive diets, e.g., vegetarian, vegan, lactose-free, etc.	Depending on the type of diet: vitamin B_12_, calcium, iron, vitamin D_3_, protein
People with known deficiencies	Taking anti-hyperglycaemic drugs—vitamin B_12_; taking methotrexate—folic acid; most of the population—vitamin D_3_
Postmenopausal women with known calcium and vitamin D deficiency	Calcium, vitamin D_3_
Infants, regardless of feeding method	Vitamin D_3_
Infants born prematurely, from multiple pregnancies, or children of mothers with anaemia during pregnancy	Iron
Newborns	One-time vitamin K intramuscular injection
Athletes	Active ingredients, protein

**Table 2:** The effect of curcumin on selected parameters of inflammation and oxidative stress.

Author and Publication Year	Cohort	Dose	Time	TAC	MDA	TNF-α	IL-6	hs-CRP	Cohen’s d * or Hedges’s g **
Hodaei et al. [18]; 2019	53 participants with T2DM, aged 40–70 years old, BMI 18.5–35 kg/m^2^	1500 mg	10 weeks	=	=	–	–	–	0 **
Jiménez-Osorio et al. [19]; 2016	101 participants with non-diabetic (*n* = 50) or diabetic (*n* = 51) proteinuric CKD, aged 20–70 years old	320 mg	8 weeks	–	↑ (8%)	–	–	–	0.16 **
Yang et al. [20]; 2015	14 participants with T2DM and CKD (no control group)	500 mg	15 days (30 days in participants with overt CKD)	–	↓↓ (~75%, compared to baseline)	–	–	–	–
Darmian et al. [21]; 2022	42 participants with T2DM and hyperlipidaemia, aged 45–60 years old	2100 mg	8 weeks	↑↑ (13%) (curcumin + aerobic training)	↓↓ (20%) (curcumin + aerobic training)	–	–	↓↓ (15%) (curcumin + aerobic training)	TAC—11.52 ** MDA—3.97 ** hs-CRP—32.45 **
				↑↑ (4%) (only curcumin)	↓↓ (8%) (only curcumin)	–	–	↓↓ (8%) (only curcumin)	TAC—3.01 ** MDA—1.24 ** hs-CRP—13.40 **
Shafabakhsh et al. [22]; 2020	60 participants with T2DM and CHD, aged 45–85 years with 2- and 3-vessel CHD	1000 mg	12 weeks	↑↑ (4%)	=	–	–	–	TAC—0.19 ** MDA—0 **
Na et al. [23]; 2014	100 overweight/obese participants with T2DM	300 mg	3 months	–	=	↓↓ (20%)	↓↓ (31%)	↓↓ (18%)	–
Adibian et al. [24]; 2019	44 participants with T2DM, aged 40–70 years old	1500 mg	10 weeks	–	–	–	–	↓↓ (15%)	0.14 **
Shafabakhsh et al. [25]; 2020	60 participants with TDM and haemodialysis, aged 18–80 years old	80 mg (nano-curcumin)	12 weeks	↑↑ (9%)	↓↓ (17%)	–	–	↓↓ (26%)	TAC—0.51 ** MDA—0.24 ** hs-CRP—22.73 **
Dastani et al. [26]; 2023	64 participants with T2DM and CAD (<70% stenosis in angiography)	80 mg (nano-curcumin)	90 days	–	–	–	–	↓↓ (28%)	0.40 *

This table presents comparison between the study group and the control group after the intervention period. T2DM—type 2 diabetes mellitus; TAC—total antioxidant capacity; MDA—Malondialdehyde; TNF-α—tumour necrosis factor α; IL-6—interleukin 6; hs-CRP—high-sensitivity C-reactive protein; CKD—chronic kidney disease; CHD—coronary heart disease; CAD—coronary artery disease; *—Cohen’s d Measure of Effect Size; **—Hedges’ g Measure of Effect Size; =—no changes; ↓↓—significant decrease; ↑—not a statistically significant increase; ↑↑—significant increase.

**Table 3:** The effect of resveratrol on selected parameters of inflammation and oxidative stress.

Author and Publication Year	Cohort	Dose	Time	TAC	MDA	TNF-α	IL-6	hs-CRP	Cohen’s d * or Hedges’s g **
Mahjabeen et al. [33]; 2022	110 participants with T2DM, aged 18–70 years old	200 mg	24 weeks	–	↓↓ (9%)	↓↓ (14%)	↓↓ (13%)	↓↓ (13%)	MDA—0.31 * TNF-α—2.5 * IL-6—0.45 * hs-CRP—22.95 *
Seyyedebrahimi et al. [34]; 2018	48 participants with T2DM, aged 30–70 years old	800 mg	2 months	↑↑ (8%)	↓ (5%)	–	–	↓ (14%)	TAC—0.3 * MDA—0.2 * hs-CRP—0.19 *
García-Martínez et al. [35]; 2023	97 older participants with T2DM	1000 mg (*n* = 37) 500 mg (*n* = 32)	6 months	↑↑ (23%) (1000 mg/d dose)	–	–	–	↓ (54%) (1000 mg/d dose)	TAC—0.98 ** hs-CRP—5.83 **
				↑ (2%) (500 mg/d dose)	–	–	–	↓ (31%) (500 mg/d dose)	TAC—0.08 ** hs-CRP—3.7 **
Ma et al. [36]; 2022	472 elderly participants with T2DM, aged >60 years old	500 mg	6 months	↓ (43%)	↓ (29%)	↓↓ (51%)	↓↓ (34%)	↓↓ (28%)	TAC—21.58 ** MDA—2.92 ** TNF-α—27.95 ** IL-6—1.47 ** hs-CRP—1.73 **
Bo et al. [37]; 2016	192 participants with T2DM, aged ≥40 years old, BMI < 35 kg/m^2^	500 mg (*n* = 65) 40 mg (*n* = 65)	6 months	–	–	–	↓ (41%) (500 mg dose)	↓ (27%) (500 mg dose)	–
				–	–	–	↓ (18%) (40 mg dose)	↓ (13%) (40 mg dose)	–
Khodabandehloo et al. [38]; 2018	45 participants with T2DM, aged 30–70 years old	800 mg	6 weeks	–	–	↓ (17%)	↓ (14%)	↓ (11%)	TNF-α—0.66 ** IL-6—0.18 ** hs-CRP—0.17 **
Bashmakov et al. [39]; 2014	24 participants with T2DM and diabetic foot syndrome	100 mg (*trans*-resveratrol)	60 days	–	–	–	–	↓ (26%)	0.20 **
Javid et al. [40]; 2019	50 participants with T2DM and chronic periodontitis, aged 30–60 years old	480 mg	4 weeks	↑ (33%)	–	↓ (3%)	↓ (16%)	–	TAC—0.62 ** TNF-α—0.36 ** IL-6—0.65 **

This table presents comparison between the study group and the control group after the intervention period; T2DM—type 2 diabetes mellitus; TAC—total antioxidant capacity; MDA—Malondialdehyde; TNF-α—tumour necrosis factor α; IL-6—interleukin 6; hs-CRP—high-sensitivity C-reactive protein; *—Cohen’s d Measure of Effect Size; **—Hedges’ g Measure of Effect Size; ↓—not a statistically significant decrease; ↓↓—significant decrease; ↑—not a statistically significant increase; ↑↑—significant increase.

**Table 4:** The effect of cinnamon on selected parameters of inflammation and oxidative stress.

Author and Publication Year	Cohort	Dose	Time	TAC	MDA	TNF-α	IL-6	hs-CRP	Cohen’s d * or Hedges’s g **
Sahib [46]; 2016	25 participants with T2DM, aged 49.1 ± 6.0 years old	1000 mg	12 weeks	–	↓↓ (33%)	–	–	–	–
Azimi et al. [47]; 2014	204 participants with T2DM, aged ≥30 years old, BMI ≥ 25 kg/m^2^	3000 mg	8 weeks	–	–	–	–	↓ (6%)	0.36 **
Davari et al. [48]; 2020	44 participants with T2DM, aged 25–70 years old	3000 mg	8 weeks	–	–	↓ (64%)	↓ (7%)	↓ (25%)	TNF-α—1.38 ** IL-6—0.43 ** hs-CRP—0.33 **
Mirmiran et al. [49]; 2019	44 participants with T2DM, aged 25–70 years old	3000 mg	8 weeks	=	=	–	–	–	–
Talaei et al. [50]; 2017	44 participants with T2DM, aged 25–70 years old	3000 mg	8 weeks	=	=	–	–	–	–

This table presents comparison between the study group and the control group after the intervention period; T2DM—type 2 diabetes mellitus; TAC—total antioxidant capacity; MDA—Malondialdehyde; TNF-α—tumour necrosis factor α; IL-6—interleukin 6; hs-CRP—high-sensitivity C-reactive protein; *—Cohen’s d Measure of Effect Size; **—Hedges’ g Measure of Effect Size; =—no changes; ↓—not a statistically significant decrease; ↓↓—significant decrease.

## Data Availability

Not applicable.
